# Single-centre review of the management of intra-thoracic oesophageal perforation in a tertiary oesophageal unit: paradigm shift, short- and long-term outcomes over 15 years

**DOI:** 10.1007/s00464-022-09682-0

**Published:** 2022-10-07

**Authors:** Vasileios Charalampakis, Victor Roth Cardoso, Alistair Sharples, Maha Khalid, Luke Dickerson, Tom Wiggins, Georgios V. Gkoutos, Olga Tucker, Paul Super, Martin Richardson, Rajwinder Nijjar, Rishi Singhal

**Affiliations:** 1 Upper GI Unit, University Hospital Birmingham NHS Foundation Trust, Birmingham, UK; 2 Institute of Cancer and Genomic Sciences, University of Birmingham, Birmingham, UK; 3 Health Data Research UK Midlands, Birmingham, UK; 4 NIHR Biomedical Research Centre, Birmingham, B15 2TT UK; 5 NIHR Surgical Reconstruction and Microbiology Research Centre, Birmingham, B15 2TT UK; 6 Athens Medical Center, Athens, Greece; 7 Department of Upper GI and Bariatric Surgery, University Hospital of North Midlands, Stoke-on-Trent, UK; 8 Liverpool University Hospitals NHS Foundation Trust, Liverpool, UK; 9 Consultant Bariatric and Upper GI Surgeon, Birmingham Heartlands Hospital, University Hospital Birmingham NHS Foundation Trust, Birmingham, UK

**Keywords:** Oesophageal perforation, Iatrogenic perforation, Boerhaave’s

## Abstract

**Background:**

Oesophageal perforation is an uncommon surgical emergency associated with high morbidity and mortality. The timing and type of intervention is crucial and there has been a major paradigm shift towards minimal invasive management over the last 15 years. Herein, we review our management of spontaneous and iatrogenic oesophageal perforations and assess the short- and long-term outcomes.

**Methods:**

We performed a retrospective review of consecutive patients presenting with intra-thoracic oesophageal perforation between January 2004 and Dec 2020 in a single tertiary hospital.

**Results:**

Seventy-four patients were identified with oesophageal perforations: 58.1% were male; mean age of 68.28 ± 13.67 years. Aetiology was spontaneous in 42 (56.76%), iatrogenic in 29 (39.2%) and foreign body ingestion/related to trauma in 3 (4.1%). The diagnosis was delayed in 29 (39.2%) cases for longer than 24 h. There was change in the primary diagnostic modality over the period of this study with CT being used for diagnosis for 19 of 20 patients (95%). Initial management of the oesophageal perforation included a surgical intervention in 34 [45.9%; primary closure in 28 (37.8%), resection in 6 (8.1%)], endoscopic stenting in 18 (24.3%) and conservative management in 22 (29.7%) patients. On multivariate analysis, there was an effect of pathology (malignant vs. benign; *p* = 0.003) and surgical treatment as first line (*p* = 0.048) on 90-day mortality. However, at 1-year and overall follow-up, time to presentation (≤ 24 h vs. > 24 h) remained the only significant variable (*p* = 0.017 & *p* = 0.02, respectively).

**Conclusion:**

Oesophageal perforation remains a condition with high mortality. The paradigm shift in our tertiary unit suggests the more liberal use of CT to establish an earlier diagnosis and a higher rate of oesophageal stenting as a primary management option for iatrogenic perforations. Time to diagnosis and management continues to be the most critical variable in the overall outcome.

**Supplementary Information:**

The online version contains supplementary material available at 10.1007/s00464-022-09682-0.

Oesophageal perforation is a surgical emergency and is associated with high morbidity and mortality with patients presenting at all hospitals and endoscopy departments. The increasing use of therapeutic endoscopy has resulted in a rise in the incidence of oesophageal perforation and these iatrogenic perforations now outnumber the more classical, spontaneous perforations (Boerhaave’s) in most recent series [1–3]. Historically, mortality rates of 13–24% have been reported with this rising further in delayed diagnosis [1–5]. There is no uniform treatment strategy sufficient to deal with all cases [6]. The choice of treatment varies depending on the aetiology of the perforation, the location and extent of the defect, the general condition of the patient, the time to presentation and local surgical expertise. The time interval between the perforation occurring and initiation of treatment is generally considered to be the most important factor that determines morbidity and mortality [2–5].

In the UK, oesophageal surgery has evolved significantly over the last decade. The improvement in outcomes in higher volume units has driven the centralisation of oesophageal services and thus the surgical management of oesophageal perforations [7, 8]. Oesophageal surgery is now performed by specialist upper gastrointestinal (UGI) surgeons rather than thoracic surgeons, as might have been the case in the past [7, 8].

A number of case series of the management of this condition have been previously published in the literature but only a few of these have been in the last decade and therefore do not necessarily reflect modern practice in a tertiary referral centre [1, 2, 5]. Much of the focus in recent years has been on the use of endoscopic stenting techniques and a number of studies have demonstrated good results using these techniques [9–12].

The aim of this study was to review our experience of managing this difficult condition in a large tertiary centre, highlighting the effect of early referral and management and reflect on the transition in the management of oesophageal perforation towards a care provided by specialised team of gastrointestinal surgeons coupled with access to minimally invasive options.

## Materials and methods

This was a single-centre, retrospective clinical review of consecutive patients treated for a perforation of the intra-thoracic oesophagus between January 2004 and December 2020. Data were collected from electronic and paper clinical records and included demographic data, method and timing of diagnosis, aetiology and location of the perforation, managing speciality, subsequent treatment, morbidity and mortality, and length of hospital stay (LOS). Data were collated for 90-day, 1-year and overall outcomes. All patients had at least 12 months of follow-up data. Iatrogenic injuries of the intra-thoracic oesophagus incurred during endoscopic procedures were included. Patients with purely cervical or abdominal perforations were excluded. Patients suffering from an anastomotic leak or an iatrogenic perforation following an open, laparoscopic or thoracoscopic surgical procedure were also excluded. This was an audit of outcomes and thus formal ethical approval was not required (http://www.hra-decisiontools.org.uk/research/). The audit was registered at Birmingham Heartlands Hospital, UK (ID number: 5197).

Continuous data were presented as mean ± standard deviation. Independent *t* test or Mann–Whitney *U* test was used to examine differences between continuous variables depending on data distribution. Chi-Square test/ Fisher’s exact test were used to compare categorical variables. Statistical analyses were performed in R. Hazard ratio were used to compare the probability of events. Survival analysis was performed using the *survival* package, data visualisation using *ggplot2* [13–15]*.*

## Results

### Demographics

Seventy-four patients were diagnosed with an intra-thoracic oesophageal perforation during the study period that satisfied all inclusion criteria. The detailed characteristics of the cohort are shown in Table 1. Forty-three patients (58.1%) were male and the mean age of the cohort was 68.28 ± 13.67 years. The overall mean follow-up available was 2.88 ± 4.12 years.

**Table 1:** Characteristics of the included cases

	Number (%)
Gender	
Male	43 (58.1)
Female	31 (41.8)
Median age	69
In-hospital mortality	20 (27)
Mean LOS (days)	42.56 ± 31.12
Speciality of care	
Thoracic	35 (47.3)
UGI	36 (48.6)
UGI + Thoracic	3 (4.1)
Location	
Upper	8
Mid	10
Distal	56
Aetiology	
Spontaneous	41 (55.4)
Iatrogenic	29 (39.2)
Trauma	1 (1.4)
Foreign body ingestion	3 (4.1)
Oesophageal pathology	
Normal	51 (68.9)
Malignant	13 (17.6)
Benign^a^	10 (13.5)
Time to presentation	
Less than 24 h	45 (60.8)
Greater than 24 h	29 (39.2)
Management	
Surgery	34 (45.9)
Stent	18 (24.3)
Conservative	22 (29.7)

^a^4 * hiatus hernia (< 5 cm), 4 * reflux oesophagitis, 2 * oesophageal candidiasis

### Diagnosis of oesophageal perforation

Twenty-two (52.38%) of the spontaneous perforations were diagnosed within 24 h as opposed to 20 (47.6%) after 24 h. However, 22 (75.9%) of the iatrogenic perforations were diagnosed within 24 h as opposed to later 7 (24.1%).

The pre-operative diagnostic modalities included CT scan in 45 (60.8%) of cases, water-soluble contrast in 20 (27%) of cases, oesophagogastroduodenoscopy in 8 (10.8%) of cases and at surgery in one (1.4%) of cases. There was no relationship between time to diagnosis and imaging modality used (*p* = 0.093). There was change in the primary diagnostic modality with CT being used for diagnosis for 19 of 20 patients (95%) after 2013 (inclusive).

### Overall management

Thirty-five (47.3%) patients were managed by thoracic surgeons, 36 (48.6%) by UGI surgeons and 3 (4.1%) were under joint thoracic and UGI care. Initial management of the oesophageal perforation included surgery in 34 (45.9%; primary closure in 28 of the 34 patients and oesophageal resection in 6 patients), endoscopic stenting in 18 (24.3%) and conservative management in 22 (29.7%) patients. There was no relationship between time to diagnosis and the first line of treatment (*p* = 0.487).

Further interventions were required in patients who initially underwent surgical management in the form of stent insertion (4 out of 34 patients; 11.76%), feeding gastrostomy insertion (1 patient; 2.9%), CT-guided drainage of left subpneumonic collection (1 patient; 2.9%) and rigid bronchoscopy, video-assisted thoracoscopic surgery, debridement and washout (1 patient; 2.9%). Similarly for patients who initially underwent stent insertion, one patient required re-stenting due to stent migration. Two patients who initially underwent a stent insertion required thoracotomy, debridement and decortication due to ongoing sepsis. Two patients initially managed conservatively subsequently required thoracotomy and primary repair due to ongoing sepsis. Of patients who required a change in treatment modality, one patient died whilst in hospital (1 of 8; 12.5%).

### Effect of aetiology on management strategy

Aetiology was spontaneous in 42 (56.76%), iatrogenic in 29 (39.2%) and foreign body ingestion/related to trauma in 3 (4.1%). Fifty-one (68.9%) patients had no underlying oesophageal pathology, 13 (17.6%) patients had an associated oesophageal malignancy and 10 (13.5%) had benign pathology. The most common initial modality of management within the spontaneous group was surgical—24 (57.1%) as opposed to the iatrogenic group where the majority underwent an endoscopic stent procedure—13 (44.8%). This difference was statistically significant (*p* = 0.013; Fisher’s exact test). Similarly, patients with malignant pathology were more likely to undergo a stenting procedure—7 out of 13 (53.8%). For those with benign pathology (*n* = 10) stenting procedure was performed in 3 (30%), surgery in 4 (40%) and conservatively in 3 (30%). Those with a normal oesophagus (*n* = 51) stenting was performed in 8 (15.7%) and surgery in 28 (54.9%). Surgery was also the most common operative modality for benign pathology/normal oesophagus (*p* = 0.035; Fisher’s exact test). Endoscopic perforation was more likely in a diseased oesophagus benign pathology—8 (80%) or malignant pathology—10 (76.9%) cases as compared to a normal oesophagus. This was statistically significant (*p* < 0.001; Fisher’s exact test).

### Length of stay

The mean length of stay was 42.56 ± 31.12 days. This was not affected by time to diagnosis (*p* = 0.078; Mann–Whitney *U* test) or initial treatment modality (*p* = 0.408; Kruskal–Wallis test).

### Mortality

In-hospital and one-year mortality was 27% (*n* = 20) and 44.6% (*n* = 33), respectively. One-year mortality was higher in patients who were diagnosed more than 24 h later—62.1% (*n* = 18/29) as opposed to if they were diagnosed within 24 h—33.3% (*n* = 15/45) (*p* = 0.014). Expectantly, 1-year mortality was higher in patients with a malignant pathology—76.1% (*n* = 10/13) as opposed to patients with a benign pathology or a normal oesophagus (37.7%, *n* = 23/61) (*p* = 0.033). Neither the aetiology of perforation (*p* = 0.298) or initial management (*p* = 0.052) affected one-year mortality.

**Fig. 1:**
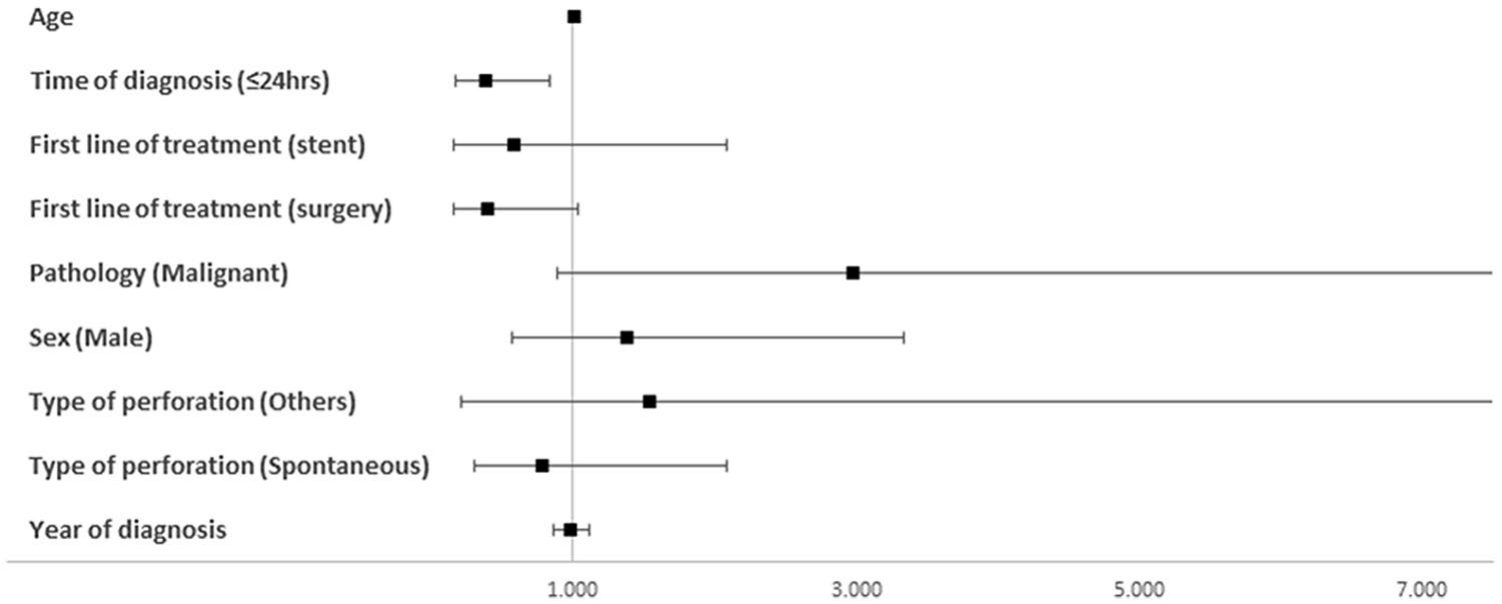
Multivariate analysis to show effect of peri-operative variables on one-year survival

On multivariate analysis (Fig. 1), there was a statistically significant effect of pathology (malignant vs. benign; *p* = 0.003) and surgical treatment as first line (*p* = 0.048) on 90-day mortality. However, this significance was no longer seen at 1-year or overall follow-up. Time to presentation (≤ 24 h vs. > 24 h) was the only significant variable (*p* = 0.017 & *p* = 0.02, respectively).

### Mortality over time

There was a trend towards improvement in 90-day mortality over time (*p* = 0.053). This trend became statistically significant for 1-year survival [HR = 0.9 (0.83–0.99); *p* = 0.028]. Patients who presented with an oesophageal perforation prior to 2010 (not inclusive), had a higher one-year mortality than subsequent patients [HR 2.35 (95% CI 1.15–4.79); *p* = 0.018].

### Survival analysis

**Fig. 2:**
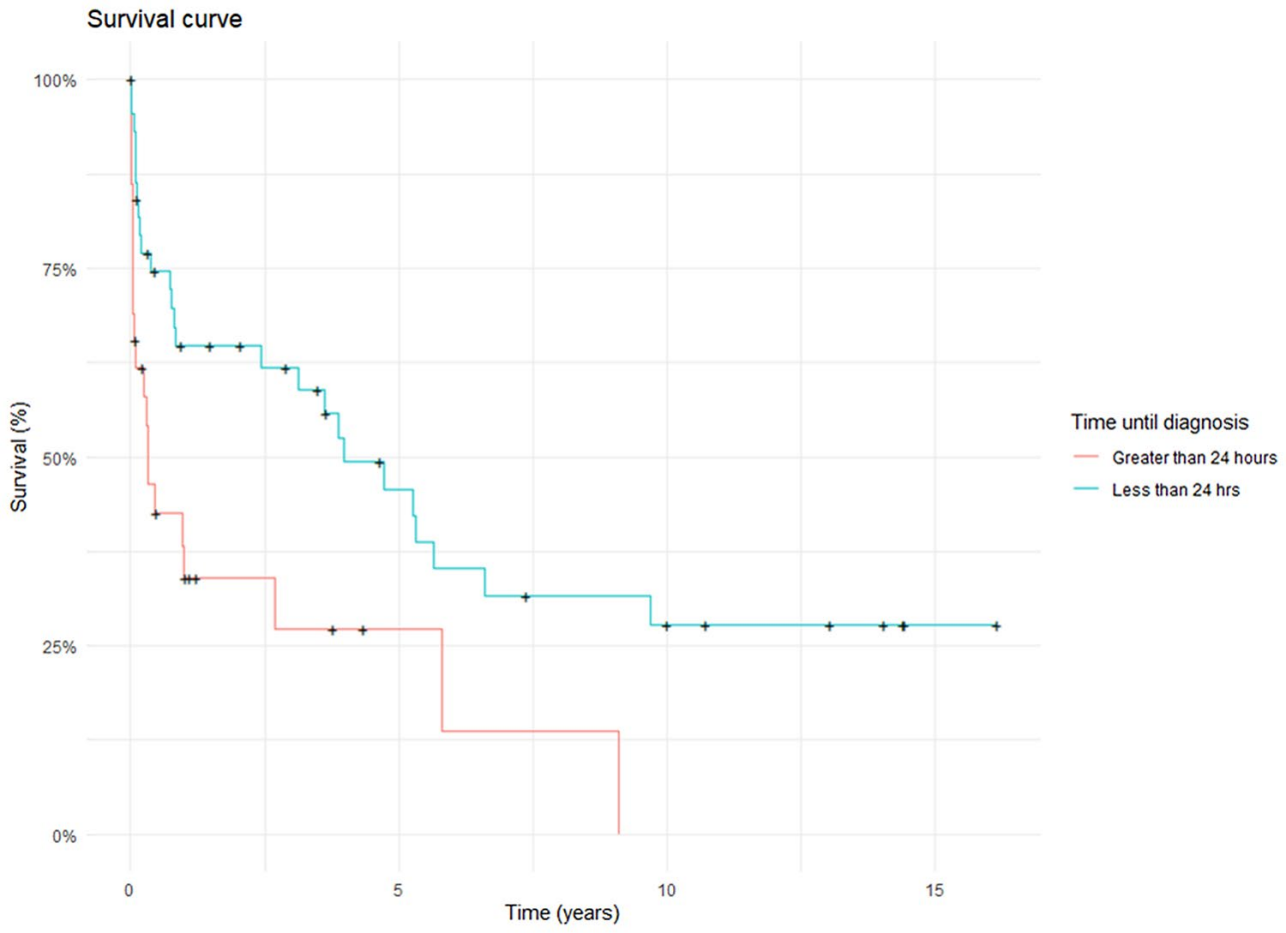
Kaplan–Meier curve comparing survival of patients diagnosed within < 24 h as opposed to those diagnosed > 24-h post-perforation

**Fig. 3:**
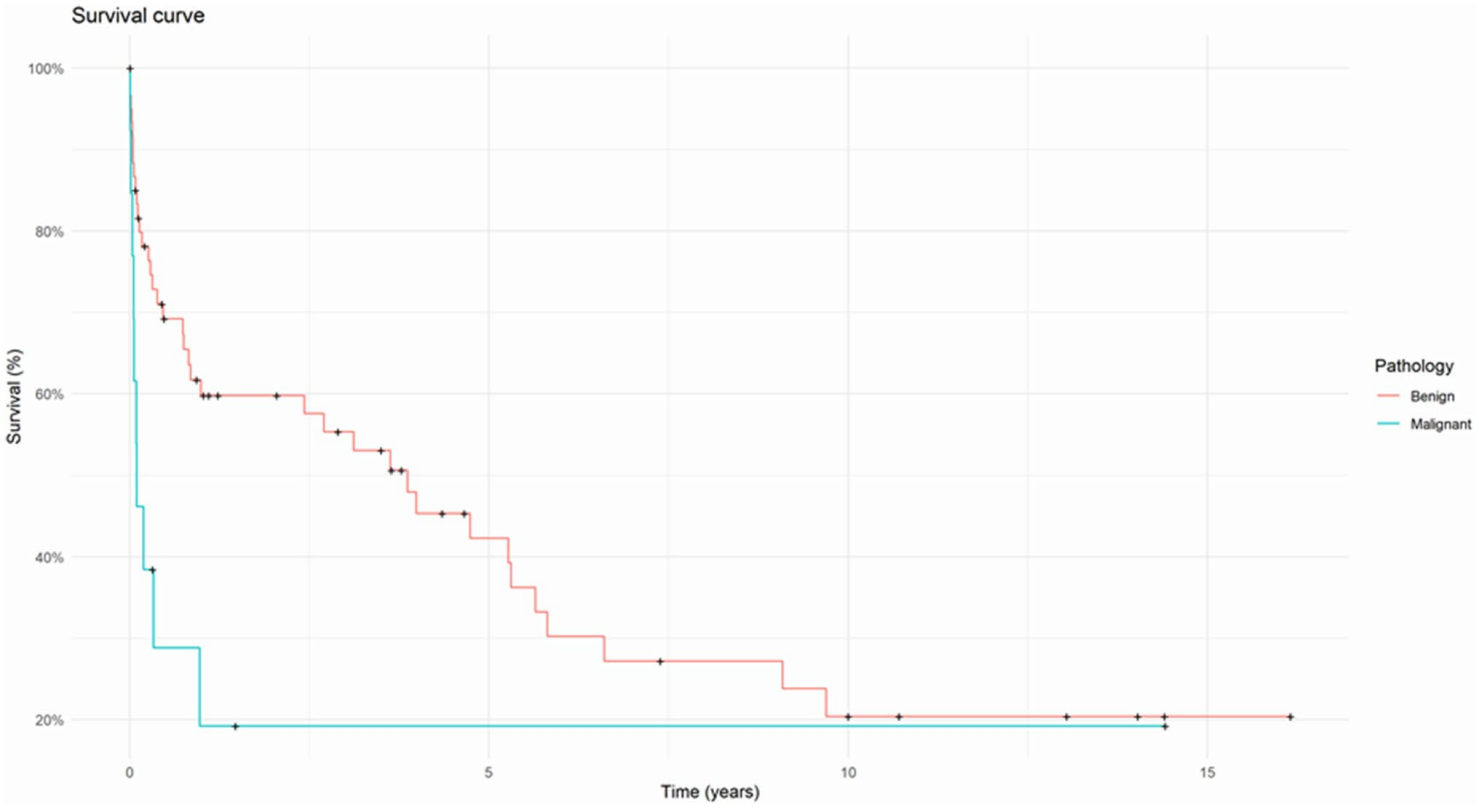
Kaplan–Meier curve comparing patients with benign pathology as opposed to those with an underlying malignancy

The effect of peri-operative variables on survival is shown in Table 2. Overall mean survival was 5.09 ± 0.83 years. Mean survival for patients who were diagnosed within 24 h was 6.47 ± 1.09 years vs. 2.37 ± 0.72 years for the patients diagnosed more than 24 h following perforation. This difference was statistically significant (*p* = 0.01, Fig. 2). Similarly, survival in patients with a malignant pathology was 3.27 ± 1.87 years vs. 5.55 ± 0.91 years for the patients with benign pathology. This difference was also statistically significant (*p* = 0.01, Fig. 3). The treating speciality (Upper GI vs Thoracic) also did not have any effect on survival (Supplementary Fig. 1).

**Table 2:** Effect of peri-operative variables on survival

		Numbers	1-year Mortality (%)	Survival (years ± SD)	P value (fisher’s exact test)
Time to diagnosis	≤ 24 h	45	33.3	6.47 ± 1.09	0.01
	> 24 h	29	62	2.37 ± 0.72	
Cause	Iatrogenic	29	55.2	3.65 ± 1.11	0.15
	Spontaneous/others	45	37.7	6.02 ± 1.13	
Pathology	Normal/benign	61	37.7	5.55 ± 0.91	0.01
	Malignant	13	76.9	3.27 ± 1.87	
Year	< 2010	36	58.3	4.85 ± 1.18	0.03
	≥ 2010	38	31.6	4.64 ± 1.02	
Managing speciality	Thoracic	35	48.6	6.22 ± 1.29	0.36
	UGI	36	44.4	3.46 ± 0.86	
	UGI + thoracic	3	0	4.74 ± 0	
Initial treatment	Conservative	22	59	4.11 ± 1.33	0.05
	Surgery	34	29.4	6.86 ± 1.36	
	Stent	18	55.6	2.06 ± 0.56

## Discussion

The current manuscript is the largest case series of intra-thoracic oesophageal perforation to be published from the UK. The current study shows that intra-thoracic oesophageal perforation continues to be associated with high in-hospital and one-year mortality. The choice of primary diagnostic modality has changed to a CT scan over the last 15 years. There was a trend towards improvement in 90-day mortality over time and this trend became statistically significant for 1-year survival. The pathology of perforation and use of surgery as first-line treatment affect short-term survival only. However, time to diagnosis and management continues to be the only variable that affects long-term survival.

Oesophageal perforation is an uncommon and difficult condition to manage. It is associated with high morbidity and mortality, although there is evidence that outcomes have improved in recent years [2]. The most common cause of oesophageal perforation described in contemporary literature is iatrogenic due to increasing use of therapeutic endoscopic techniques such as stenting and dilatation [16]. Perforation is a rare complication of diagnostic endoscopy but is more common when therapeutic dilatation or other modern complex endoscopic procedures are employed [17].

There are a number of factors accounting for the high mortality associated with oesophageal perforation. It is uncommon and presents often with non-specific symptoms such as vomiting, epigastric and chest pain. A high index of suspicion is required to establish the diagnosis early as these symptoms can be attributed to a number of common pathologies. Diagnosis is often delayed and this can have a significantly deleterious impact on subsequent outcomes [2, 5]. In addition, the oesophagus is a notoriously difficult organ upon which to operate. It is relatively inaccessible, traverses three body cavities, has a fragile blood supply coupled with lack of a serosal layer. Even under optimal conditions, oesophageal surgery is associated with significant morbidity and mortality. Finally, patients who suffer this unfortunate condition are often elderly and frail and often lack the physiological reserve to tolerate the physiological impact of both the conditions and the subsequent treatment. This can be compounded by the presence of an underlying malignancy as found in 17.6% of our population.

The in-hospital mortality in the current series was 27%. This is comparable to mortality rates reported in the previous series [3, 16, 18]. Mortality rates more than doubled when the diagnosis was delayed for greater than 24 h. Mortality rates were not affected by aetiology of perforation, despite patients with iatrogenic perforations being more likely to present early. Given the importance of early diagnosis, it may be expected that thus this group should see a survival advantage over spontaneous perforations. Indeed, many other authors have demonstrated a lower mortality in patients with iatrogenic perforations [1, 16, 19]. However, the mortality in this group is likely to be increased as a result of underlying pathology. Iatrogenic perforations were more common in patients with an underlying pathology (18 of 29; 62%).

The current series has a higher number of spontaneous perforations in comparison to recent literature as it covers a period of more than 15 years and may reflect the referral pathways necessitated by centralisation of oesophageal surgery in the UK. Spontaneous perforations represent a minority of cases in most recent series [3, 16, 20].

A number of different management pathways are common in clinical practice for treating oesophageal perforations. Traditionally, surgical management has been considered the only viable option due to the almost universal mortality associated with non-surgical management in the pre-antibiotic era. Indeed, in the current series, surgical repair remains the most common management strategy. 45.9% of patients underwent initial surgical management (including six oesophagectomies) and a further 5.4% (4 of 74) required surgery after failed attempts at conservative management or endoscopic stenting. Surgery, therefore remains an important part of the management algorithm despite the increasing popularity of less invasive strategies.

Many authors have reported excellent results from primary repair of oesophageal perforation [2, 20, 21]. Good results have even been described in patients diagnosed late [19]. The overall operative mortality (17.6%) in the current series is comparable to that reported in the literature.[3, 22]. Interesting, even for patients diagnosed later than 24 h after symptom onset; mortality after surgery was 25%; less than the in-hospital mortality of 27%.

We analysed the changes in primary diagnostic modality over time. Historically, water-soluble contrast swallow oesophagogram has been considered the gold-standard diagnostic test and was used extensively in the early part of our study. However, CT has been shown to be equally, if not more effective as a diagnostic modality and offers significant advantages over the oesophagogram as it is able to identify other pathologies and to assess the extent of air and fluid collections in the mediastinum and pleural cavities [23].

The highest in-hospital mortality was seen with conservative treatment (10 of 22 patients; 45.5%). Although the successful management of iatrogenic and even spontaneous, oesophageal injuries with conservative management has been described; this approach can be associated with high morbidity and mortality. [24–27] A confounding factor is that this group might include patients who were deemed physiologically unfit for surgery due to their poor condition and/or background health.

In contrast, good results have been shown for the use of covered oesophageal stents by multiple authors [9–12]. Placement of a covered stent allows for the precise restoration of luminal integrity and will prevent further mediastinal contamination. Stents have also been used to manage spontaneous perforations but with less success [28, 29]. This is because a stent is usually not able to control established mediastinal and pleural sepsis and therefore may require a combination with a drainage procedure. This makes their use for iatrogenic perforation more promising, as mediastinal soiling is less likely in the starved patient. Moreover, iatrogenic perforations tend to be diagnosed before any major contamination occurs. In the current series, stenting was predominantly used for managing iatrogenic perforations (13 of 18 stents were placed for iatrogenic perforation) and was successful in sealing the perforation in all patients.

The provision of oesophageal surgery has changed drastically over the last decade in the UK. Oesophageal surgery is no longer performed by thoracic surgeons but instead GI surgeons, specialised in the Upper GI Tract [30]. These changes are reflected in our data. There was a notable reduction in the one-year mortality for surgical patients during the course of our study period. Similar improvements in mortality have been seen for patients undergoing elective oesophagogastric surgery for oesophagogastric cancer [31]. It is likely that increased specialisation and the centralisation of oesophagogastric services has resulted in the creation of systems whereby these complex patients being managed by specialist teams who possess the necessary medical, surgical and endoscopic skills. High-volume oesophageal centres have all diagnostic and treatment modalities readily available and so the treatment is dictated by necessity rather than availability.

This study has some limitations. It is a retrospective review from a single centre and therefore is open to data collection bias. The number of patients is small, however, oesophageal perforations are unusual and subsequently larger numbers are not easy to achieve. Also, though a higher mortality was noticed for patients with an oesophageal perforation on a background of malignancy, it was impossible to be certain of the eventual cause of death since routine follow-up imaging was not performed for these patients. On the other hand, the strength of our study is that it presents clear evidence that time to diagnosis is an important prognostic factor in long-term survival from intra-thoracic perforations. It also demonstrates a paradigm shift in the management of oesophageal perforation within the same hospital and setting, subsequently reducing other confounding factors and demonstrating the effect of specialisation and minimal invasive approach to the outcomes.

## Conclusion

Oesophageal perforation remains a condition with a high mortality due to the difficulties in making an early diagnosis, the variety of underlying pathologies and the complexity of management pathways. Trends in our unit suggest that the more liberal use of CT to establish an earlier diagnosis, a higher rate of oesophageal stenting as a primary management option for iatrogenic perforations and centralisation of oesophageal services have been associated with an improvement in overall patient outcomes.

## Supplementary Information

Below is the link to the electronic supplementary material.

Supplementary Figure 1: Kaplein-Meier Curve comparing survival of patients who were treated by Upper GI surgeons as opposed to Thoracic surgeons. (TIFF 2631 KB)
