# Supplementary figures and images for: Single-centre review of the management of intra-thoracic oesophageal perforation in a tertiary oesophageal unit: paradigm shift, short- and long-term outcomes over 15 years
Source: Surg Endosc. 2022 Oct 7;37(3):1710–7. doi: 10.1007/s00464-022-09682-0 (PMC10017567; doi:10.1007/s00464-022-09682-0)

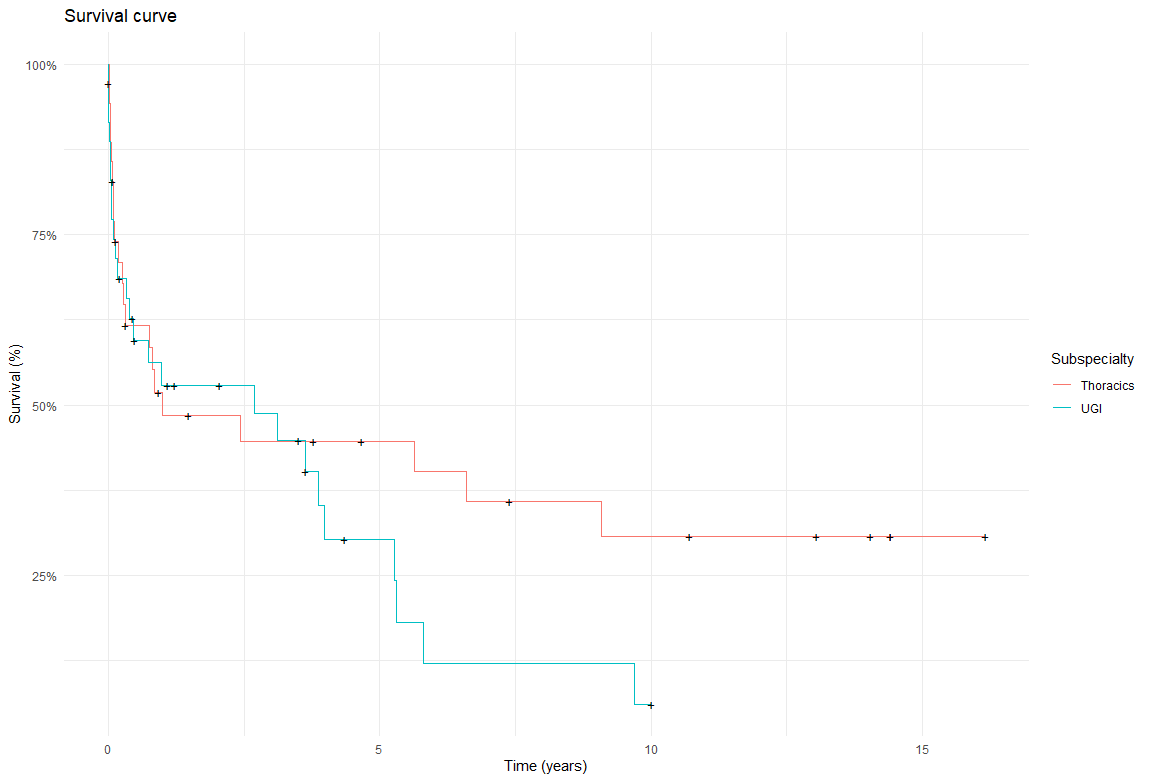

Supplement: Supplementary file 1 — Supplementary Figure 1: Kaplein-Meier Curve comparing survival of patients who were treated by Upper GI surgeons as opposed to Thoracic surgeons. (TIFF 2631 KB) [file 464_2022_9682_MOESM1_ESM.tiff]
